# Supplementary material for: Knowledge and occupational practices of beauticians and barbers in the transmission of viral hepatitis: A mixed-methods study in Volta Region of Ghana
Source: PLoS One. 2025 Jan 16;20(1):e0306961. doi: 10.1371/journal.pone.0306961 (PMC11737681; doi:10.1371/journal.pone.0306961)
Supplement: Attachment — Submitted filename: Viral hepatitis review_plosOne.docx [file pone.0306961.s003.docx]

**Reviewer’s comments**

**Manuscript ID:** PONE-D-24-25615

**Title: Knowledge and occupational practices of beauticians and barbers in the transmission of viral hepatitis: a mixed-methods study in Volta Region of Ghana**

**General comments**

Given the enormity of the impact of viral hepatitis on quality of life, morbidity and mortality, this topic remains relevant, and therefore, I wish to commend that authors for a good job done. The authors are however advised to meticulously proofread their work and address a few specific comments.

**Specific comments**

**Abstract**

**Background**

1. The opening sentence should be revised as it appears not to articulate the problem. The authors can consider stating the problem as “Hepatitis B and C viral infections are endemic in Ghana. Also, the National Policy on Viral Hepatitis stipulates that there is unreliable data, limited knowledge, and deficiency in research on viral hepatitis, especially among high risk workers in the eastern part of the country”

**Methods**

2. Please break this sentence into two to read well: “While an in-depth interview was used to collect data from five environmental health officers who were selected as key informants in the qualitative stage, structured questionnaires/checklists, and direct observations were employed to collect data from 340 beauticians and barbers in the quantitative stage”.

**Conclusion**

3. The conclusion sub-section of the abstract is good except that I recommend that, the authors add a brief sentence on recommendations regarding how the problem can be addressed.

**Introduction**

4. Lines 116-117: Please provide a citation to the statement “The ability to prevent HBV and HBC infections among beauticians and barbers as well as their customers is highly dependent on having adequate knowledge about these infections.”
5. Lines 128-129: Please provide relevant citation to “…however, other practical routes of infection including the operations of beauticians and barbers could be a source of transmission”

**Methods**

**Study area**

6. The authors have aptly given a brief profile of the study area. However, I recommend that the authors write on the healthcare delivery systems in the study area, including specialized arrangements for hepatitis B and C testing if available.

**Sample size determination**

7. The authors estimated the sample size of the quantitative arm of the study using a design effect size = 1.5. Can the authors justify why such design effect size was chosen? What was the guiding principle?
8. The authors did not mention how the sample size for the qualitative arm of the study was obtained. Authors are advised to address it.

**Data collection and variables**

9. How were the questionnaire, interview guide and checklist designed? What was the content of each instrument? Was the questionnaire validated and tested for reliability? The authors are strongly advised to elaborate on the data collection instruments**.**
10. The authors failed to explicitly state the variables of the study. For the purposes of clarity, authors are advised to clarify what variables were being used in their study.

**Statistical analysis**

11. Lines 245-247: What guided the use of median as cut-off point? Did the authors visualize the data? Was the data normally distributed or not. Please elaborate your choice of median as the measure of central tendency. As a guide, use mean as measure of central tendency for normally distributed data and median for skewed data.
12. Referring to Tables 4 & 5, it is obvious that some cells of the contingent tables have fewer counts. Consequently, biased estimates have been obtained as large odds ratios and wide confidence intervals. I strongly advise that the authors account for such sparse data which have produced large odds ratios and wide confidence intervals. Authors may address this concern by referring to Balegha (2024). <https://doi.org/10.1371/journal.pgph.0003160>; Heinz (2010). <https://doi.org/10.1002/sim.3794> and Firth (1993). <https://doi.org/10.1093/biomet/80.1.27>

**Discussion**

13. The authors attempted a great discussion of the study findings. However, the authors have not adequately espoused and discussed similar and contrasting findings of previous studies. Therefore, the authors could not elaborate on the practical and policy implications of the results. I therefore recommend that the authors thoroughly discuss their findings taking note of similar findings, contrasting findings, explanation for such discrepancies, the practical import of the findings, theoretical and policy aspects of the results if any.
