# Supplementary material for: Knowledge and occupational practices of beauticians and barbers in the transmission of viral hepatitis: A mixed-methods study in Volta Region of Ghana
Source: PLoS One. 2025 Jan 16;20(1):e0306961. doi: 10.1371/journal.pone.0306961 (PMC11737681; doi:10.1371/journal.pone.0306961)
Supplement: Attachment — Submitted filename: Review_result_PONE-D-24-25615.docx [file pone.0306961.s004.docx]

**Topic: Knowledge and Occupational Practices of Beauticians and Barbers in the Transmission of Viral Hepatitis: A Mixed-Methods Study in the Volta Region of Ghana**

**Manuscript Number:** PONE-D-24-25615
**Reviewer:** ZB

**Detailed Comments**

**Background**

**Lines 145-147:** The original text states: “Furthermore, there is unreliable data, limited knowledge, and a deficiency in research with no established occupational safety regulations on viral hepatitis prevention and control, especially in the Volta region of Ghana (1).” This suggests that the study is focused on policy and regulation issues. Please revise this to align with the study's intention, which is to investigate knowledge and practices regarding viral hepatitis.

**Methods**

**Sampling:**

- **Statement:** “To recruit the 340 beauticians and barbers, five districts’ capital towns were selected using simple random sampling at the initial stage.”
  **Comment:** The explanation of how simple random sampling was used to recruit respondents is unclear. Please specify the sampling unit.
- **Population Clarification:** Clarify the term “the population size of each selected town.” Does this refer to the resident population or the population targeted by the study?
- **Snowball Sampling:** The use of a snowball sampling procedure from an “available member list” to select participants needs clarification. Why was this technique used instead of obtaining a list of beauticians and barbers, which are identifiable business activities? How was the sample distributed between beauticians and barbers?

**Qualitative Component:**

- **Sample Size:** Explain how the sample size for the qualitative component was determined. Include details about the qualifications and training of data collectors. Also, clarify the duration of each interview, as 20 minutes seems too short and potentially unproductive.

**Observation:**

- **Sample Determination:** How was the observation sample determined? Were observations conducted over different days of the week? Address how social desirability bias was managed, considering that respondents were aware of being observed.

**Analysis:**

- **Knowledge and Practice Scores:** The method for estimating absolute knowledge and practice scores using a median cut-off point is confusing. How do Cronbach’s alpha coefficients of 0.81 and 0.79 relate to establishing a cut-off point for knowledge scores? Please revisit and clarify this.

**Qualitative Analysis:**

- **Coding and Triangulation:** Clarify whether the analysis was manual or software-assisted. Explain how the code structure was defined and established, the number of coders involved, and how agreement was achieved among them. Additionally, explain how the results were triangulated with the quantitative findings.

**Data Collection Tools/Instrument:**

- **Comprehensiveness:** Include comprehensive information about the data collection tools, including validity (content-wise), the number of items, measurement methods and scoring techniques, and the operational definitions of knowledge and practices.

**Results**

- **Table 1:** The qualitative background should not be included in this table and should be presented separately.
- **Presentation of Findings:** The results from the quantitative and qualitative analyses are presented separately, with no integration of the findings. This section should be revised to integrate and triangulate both results effectively.

**Discussion**

- **Integration Required:** The discussion currently separates findings from the quantitative and qualitative analyses. This approach is incorrect. The discussion should be integrated, analyzing and discussing both sets of findings together to draw meaningful conclusions and implications. A careful revision is required in this section.
