# Supplementary material for: Knowledge and occupational practices of beauticians and barbers in the transmission of viral hepatitis: A mixed-methods study in Volta Region of Ghana
Source: PLoS One. 2025 Jan 16;20(1):e0306961. doi: 10.1371/journal.pone.0306961 (PMC11737681; doi:10.1371/journal.pone.0306961)
Supplement: Attachment — Submitted filename: Response to reviewers_PONE.docx [file pone.0306961.s005.docx]

16th September, 2024.

The Editor-in-chief,

Plos One.

**RESPONSES TO JOURNAL REQUIREMENTS AND REVIEWERS’ COMMENTS**

Many thanks for kindly reviewing our manuscript. We have carefully considered all concerns raised and incorporated feedback, where possible. The quality of the revised manuscript has greatly improved as a result of your useful comments. We have provided point-by-point responses to the respected observations and comments raised as exhibited below. Please note that the changes in the revised manuscript have been highlighted in red.

Thank you.

Yours Sincerely,

Silas Adjei-Gyamfi

(Corresponding author)

**JOURNAL REQUIREMENTS**

**[Comment 1]** When submitting your revision, we need you to address these additional requirements. Please ensure that your manuscript meets PLOS ONE's style requirements, including those for file naming. The PLOS ONE style templates can be found at 
<https://journals.plos.org/plosone/s/file?id=wjVg/PLOSOne_formatting_sample_main_body.pdf> and <https://journals.plos.org/plosone/s/file?id=ba62/PLOSOne_formatting_sample_title_authors_affiliations.pdf>

**🡺 [Response 1**] Thanks for the comment. The manuscript has been revised to meet PLOS ONE’s style.

**[Comment 2]** Thank you for stating in your Funding Statement: This study was supported by The Project for Human Resource Development Scholarship, Japan International Cooperation Agency (JICA), and Nagasaki University School of Tropical Medicine and Global Health, Japan. Please provide an amended statement that declares all the funding or sources of support (whether external or internal to your organization) received during this study, as detailed online in our guide for authors at <http://journals.plos.org/plosone/s/submit-now>.  Please also include the statement “There was no additional external funding received for this study.” in your updated Funding Statement. Please include your amended Funding Statement within your cover letter. We will change the online submission form on your behalf.

**🡺 [Response 2]** Thanks for the useful comments. The funding statement has been modified as “This study was funded by The Project for Human Resource Development Scholarship under the Japan International Cooperation Agency (JICA), and Nagasaki University School of Tropical Medicine and Global Health, Japan through MPH scholarship support which was received by FKK from 2019 to 2021. There was no additional external funding received for this study. The funders had no role in study design, data collection and analysis, decision to publish, or preparation of the manuscript”

**[Comment 3]** We are unable to open your Supporting Information file "PONE_S1 quan data.dta". Please kindly revise as necessary and re-upload.

**🡺 [Response 3]** Thanks for this comment. The supporting file “PONE_S1 quan data.dta” is the dataset for the quantitative arm of the mixed-method study which was collected and analysed for the study. The file has been revised and re-uploaded.

**[Comment 4]** Your ethics statement should only appear in the Methods section of your manuscript. If your ethics statement is written in any section besides the Methods, please delete it from any other section.

**🡺 [Response 4]** Thanks for the suggestion. The Ethics statement can only be found in the methods section (see page 13; lines 262–268; ethical considerations; materials and methods)

**Reviewer #1**

**[General comment]** Given the enormity of the impact of viral hepatitis on quality of life, morbidity and mortality, this topic remains relevant, and therefore, I wish to commend that authors for a good job done. The authors are however advised to meticulously proofread their work and address a few specific comments.
**🡺 [General Response]** We appreciate the reviewer’s deep comprehension and recognition of the significance of our manuscript. Proofreading has been done now. We are pleased to address the useful comments and suggestions made by Reviewer #1 below.

**Abstract
Background**
**[Comment 1]** The opening sentence should be revised as it appears not to articulate the problem. The authors can consider stating the problem as “Hepatitis B and C viral infections are endemic in Ghana. Also, the National Policy on Viral Hepatitis stipulates that there is unreliable data, limited knowledge, and deficiency in research on viral hepatitis, especially among high risk workers in the eastern part of the country”
**🡺 [Response 1]** Thanks for this important suggestion. Having supported your suggestion, the background of the abstract has been revised (see page 2; lines 23–25; abstract; background).

**Methods**
**[Comment 2]** Please break this sentence into two to read well: “While an in-depth interview was used to collect data from five environmental health officers who were selected as key informants in the qualitative stage, structured questionnaires/checklists, and direct observations were employed to collect data from 340 beauticians and barbers in the quantitative stage”.
**🡺 [Response 2]** Thanks for the useful suggestion. The sentence has been divided into two (see page 2; lines 32–35; abstract; methods).

**Conclusion**
**[Comment 3]** The conclusion sub-section of the abstract is good except that I recommend that, the authors add a brief sentence on recommendations regarding how the problem can be addressed.
**🡺 [Response 3]** Thanks for the comments. Having supported your suggestion, a brief statement on recommendations has been added (see page 3; lines 55–57; conclusion; abstract).

**Introduction**
**[Comment 4]** Lines 116-117: Please provide a citation to the statement “The ability to prevent HBV and HBC infections among beauticians and barbers as well as their customers is highly dependent on having adequate knowledge about these infections.”

**🡺 [Response 4]** Thanks for the comment. The above sentence has been cited (see page 6; lines 113–114; introduction).

**[Comment 5]** Lines 128-129: Please provide relevant citation to “…however, other practical routes of infection including the operations of beauticians and barbers could be a source of transmission”

**🡺 [Response 5]** Thanks for the comment. The above sentence has been referenced as suggested (see page 6; lines 125–126; introduction).

**Methods
Study area**
**[Comment 6]** The authors have aptly given a brief profile of the study area. However, I recommend that the authors write on the healthcare delivery systems in the study area, including specialized arrangements for hepatitis B and C testing if available.

**🡺 [Response 6]** Thanks for the suggestion. Healthcare delivery systems in the study area among others have been added (see pages 8–9; lines 160–172; methods and materials; study area).

**Sample size determination**
**[Comment 7]** The authors estimated the sample size of the quantitative arm of the study using a design effect size = 1.5. Can the authors justify why such design effect size was chosen? What was the guiding principle?

**🡺 [Response 7]** Thanks for the comment. The design effect used in this study was based on the WHO conservative design effect (DEFF) size of 1.5. This DEFF indicates how well a sample of street beauticians and barbers in the region represents the larger population which is employed in most hepatitis related studies. With the level of expected practices and absolute precision including 95% confidence intervals, a conservative DEFF of 1.5 was used to account for any expected increase in variance due to clustering (see page 10; line 193; methods and materials; sample size and sampling).

**[Comment 8]** The authors did not mention how the sample size for the qualitative arm of the study was obtained. Authors are advised to address it.

**🡺 [Response 8]** Thanks for the comment. Having supported your comment, qualitative studies do not usually have calculations for sample size but mostly quote the number of participants for sample size. Either a specific number of participants are interviewed or the interviews are done based on saturation during qualitative research. Hence, the qualitative arm of this study used five key informants (one from each study site). Details are explained in the manuscript (kindly see page 10; lines 213–218; methods and materials; sample size and sampling).

**Data collection and variables**
**[Comment 9]** How were the questionnaire, interview guide and checklist designed? What was the content of each instrument? Was the questionnaire validated and tested for reliability? The authors are strongly advised to elaborate on the data collection instruments.

**🡺 [Response 9]** Thanks for this useful comment. Having supported your suggestion, the data collection section has been revised in the manuscript (see page 12; lines 232–251; methods and materials; data collection and variables)

**[Comment 10]** The authors failed to explicitly state the variables of the study. For the purposes of clarity, authors are advised to clarify what variables were being used in their study.

**🡺 [Response 10]** Thanks for this comment. A table has been added to describe the key quantitative variables (see Table 2; page 15).

**Statistical analysis**
**[Comment 11]** Lines 245-247: What guided the use of median as cut-off point? Did the authors visualize the data? Was the data normally distributed or not. Please elaborate your choice of median as the measure of central tendency. As a guide, use mean as measure of central tendency for normally distributed data and median for skewed data.

**🡺 [Response 11]** Thanks for this in-depth suggestion. The knowledge and practice scores of participants were first visualized to check their distribution of which both were skewed (non-normal) distribution. Hence, the median was appropriate for the analysis. Precise elaboration is shown in the manuscript now (see page 16; lines 308–310; methods and materials; statistical analysis).

**[Comment 12]** Referring to Tables 4 & 5, it is obvious that some cells of the contingent tables have fewer counts. Consequently, biased estimates have been obtained as large odds ratios and wide confidence intervals. I strongly advise that the authors account for such sparse data which have produced large odds ratios and wide confidence intervals. Authors may address this concern by referring to Balegha (2024). <https://doi.org/10.1371/journal.pgph.0003160>; Heinz (2010). <https://doi.org/10.1002/sim.3794> and Firth (1993). <https://doi.org/10.1093/biomet/80.1.27>
**🡺 [Response 12]** Thanks for this insightful comment. The statistical analysis section has been revised to address biased estimates (see pages 16–17; lines 317–333; methods and materials; statistical analysis). Moreover, refer to Tables 7 and 8 on pages 30 and 31 respectively.

**Discussion**
**[Comment 13]** The authors attempted a great discussion of the study findings. However, the authors have not adequately espoused and discussed similar and contrasting findings of previous studies. Therefore, the authors could not elaborate on the practical and policy implications of the results. I therefore recommend that the authors thoroughly discuss their findings taking note of similar findings, contrasting findings, explanation for such discrepancies, the practical import of the findings, theoretical and policy aspects of the results if any.

**🡺 [Response 13]** Thanks for this remarkable comment. The discussion section has been carefully revised to meet the suggestions above (Kindly see pages 32–38; lines 572–705; discussion).

**Reviewer #2**

**Background**
**[Comment 1]** Lines 145-147: The original text states: “Furthermore, there is unreliable data, limited knowledge, and a deficiency in research with no established occupational safety regulations on viral hepatitis prevention and control, especially in the Volta region of Ghana (1).” This suggests that the study is focused on policy and regulation issues. Please revise this to align with the study's intention, which is to investigate knowledge and practices regarding viral hepatitis.

**🡺 [Response 1]** Thanks for the comments. The above sentence has been revised as “Besides the fact that there is unreliable data and deficient occupational safety regulations on viral hepatitis, there is also limited research on the awareness of hepatitis and work-related practices among street beauticians and barbers, especially in the Volta region of Ghana.” (see page 7; lines 142–145; introduction).

**Methods**
**Sampling:**
**[Comment 2]** Statement: “To recruit the 340 beauticians and barbers, five districts’ capital towns were selected using simple random sampling at the initial stage.”
Comment: The explanation of how simple random sampling was used to recruit respondents is unclear. Please specify the sampling unit.

**🡺 [Response 2]** Thanks for this insightful comment. Details on the selection procedure has been added to the sampling session (see page 10; lines 197–200; materials and methods; sample size and sampling).

**[Comment 3]** Population Clarification: Clarify the term “the population size of each selected town.” Does this refer to the resident population or the population targeted by the study?

**🡺 [Response 3]** Thanks for the comment. This is referring to the resident population since there is no available member list in the region. Revisions have been made now (see page 10; lines 201–203; materials and methods; sample size and sampling). Also, see Table 1 on page 11.

**[Comment 4]** Snowball Sampling: The use of a snowball sampling procedure from an “available member list” to select participants needs clarification. Why was this technique used instead of obtaining a list of beauticians and barbers, which are identifiable business activities? How was the sample distributed between beauticians and barbers?

**🡺 [Response 4]** Thanks for this comment. Snowballing sampling was used for the study because it is very difficult to locate street barbers and beauticians as they do not operate from a fixed place and there was no available member list. This technique was also useful as most of the street barbers and beauticians do not have Bussiness Operating Permit (BOT) and are evading tax payments. Therefore, referral by somebody within their social network increased their trust in participating in the study (see page 10; lines 205–210; materials and methods; sample size and sampling).

**Qualitative Component:**
**[Comment 5]** Sample Size: Explain how the sample size for the qualitative component was determined. Include details about the qualifications and training of data collectors. Also, clarify the duration of each interview, as 20 minutes seems too short and potentially unproductive.

**🡺 [Response 5]** Thanks for the beautiful comment. The manuscript has been revised to take care of the above comments. On how sample size was selected, kindly see (page 11; lines 213–218; materials and methods; sample size and sampling). For qualifications and training of data collectors, kindly see (page 12; lines 246–251; materials and methods; data collection and variables). Finally, for the duration of the interview, please see (page 14; lines 285–286; materials and methods; data collection and variables). The time excludes the preparatory and the administrative processes.

**Observation:**
**[Comment 6]** Sample Determination: How was the observation sample determined? Were observations conducted over different days of the week? Address how social desirability bias was managed, considering that respondents were aware of being observed.
**🡺 [Response 6]** Thanks for the comment. The observation sample was determined as 340 as shown in the sample size calculation. Observations were conducted over different days of the week. Finally, we preempted the possibility of social desirability bias and took several measures to reduce or mitigate its tendencies throughout the data collection process (see page 13; lines 257–268; materials and methods; data collection and variables).

**Analysis:**
**[Comment 7]** Knowledge and Practice Scores: The method for estimating absolute knowledge and practice scores using a median cut-off point is confusing. How do Cronbach’s alpha coefficients of 0.81 and 0.79 relate to establishing a cut-off point for knowledge scores? Please revisit and clarify this.

**🡺 [Response 7]** Thanks for this significant suggestion. Having supported your suggestion, clarifications have been made. The knowledge and practice scores of participants were first visualized to check their distribution of which both were skewed (non-normal) distribution. Hence, the median cut point was appropriate for the analysis as evidenced in other earlier studies (see page 16; lines 307–310; materials and methods; statistical analysis).

**Qualitative Analysis:**
**[Comment 8]** Coding and Triangulation: Clarify whether the analysis was manual or software-assisted. Explain how the code structure was defined and established, the number of coders involved, and how agreement was achieved among them. Additionally, explain how the results were triangulated with the quantitative findings.

**🡺 [Response 8]** Thanks for the comment. The above comments have been addressed. Kindly see (page 16–17; lines 335–352; materials and methods; statistical analysis)

**Data Collection Tools/Instrument:**
**[Comment 9]** Comprehensiveness: Include comprehensive information about the data collection tools, including validity (content-wise), the number of items, measurement methods, and scoring techniques, and the operational definitions of knowledge and practices.

**🡺 [Response 9]** Thanks for this information. Comprehensive information on the data collection tools have been added (see page 12; lines 232–251; materials and methods; data collection and variables). Also see Table 2: Description of key variables (see page 15; materials and methods; data collection and variables).

**Results**
**[Comment 10]** Table 1: The qualitative background should not be included in this table and should be presented separately.

**🡺 [Response 10]** Thanks for this comment. As suggested and seen in Table 3, the background of respondents for the qualitative arm has been presented separately. Kindly see Tables 3 and 4 on pages 20–21.

**[Comment 11]** Presentation of Findings: The results from the quantitative and qualitative analyses are presented separately, with no integration of the findings. This section should be revised to integrate and triangulate both results effectively.

**🡺 [Response 11]** Thanks for the comment. The findings have been integrated and triangulated (see pages 21–27; lines 400–521; results)

**Discussion**
**[Comment 12]** Integration Required: The discussion currently separates findings from the quantitative and qualitative analyses. This approach is incorrect. The discussion should be integrated, analyzing and discussing both sets of findings together to draw meaningful conclusions and implications. A careful revision is required in this section.

**🡺 [Response 12]** Thanks for this significant comment. The discussion section has been carefully revised to meet the suggestions above (Kindly see pages 32 –38; lines 572–705; discussion).
