# Supplementary material for: Knowledge and occupational practices of beauticians and barbers in the transmission of viral hepatitis: A mixed-methods study in Volta Region of Ghana
Source: PLoS One. 2025 Jan 16;20(1):e0306961. doi: 10.1371/journal.pone.0306961 (PMC11737681; doi:10.1371/journal.pone.0306961)
Supplement: Attachment — Submitted filename: Final comment_Beauticians & Barbers_Viral hep.docx [file pone.0306961.s006.docx]

**Reviewer 1 comment on revised manuscript**

**Full title:** Knowledge and occupational practices of beauticians and barbers in the transmission of viral hepatitis: a mixed-methods study in Volta Region of Ghana

**Manuscript ID:** PONE-D-24-25615R1

**Comment**

I wish to congratulate the authors for a great job done in addressing the comments raised in the original manuscript.

However, the authors are encouraged to address comment 8 of the previously mentioned- on the guiding principle for the number of study participants selected in the qualitative arm of the study.

As rightly pointed out by the authors there is no specific formula for sample size determination in qualitative research except for the principle of data saturation. I think that the authors should state in the manuscript that data saturation was used as the guiding principle for recruitment.

Once again congratulations!!!
