# Supplementary material for: Knowledge and occupational practices of beauticians and barbers in the transmission of viral hepatitis: A mixed-methods study in Volta Region of Ghana
Source: PLoS One. 2025 Jan 16;20(1):e0306961. doi: 10.1371/journal.pone.0306961 (PMC11737681; doi:10.1371/journal.pone.0306961)
Supplement: Attachment — Submitted filename: Response to reviewers_PONE.docx [file pone.0306961.s007.docx]

Dear Editor-in-chief,

**RESPONSE TO REVIEWERS’ COMMENTS**

Many thanks for the second and rigorous review of our manuscript. We have carefully addressed all concerns raised and provided feedback, where possible. Due to your significant comments, the quality of the revised manuscript has been enormously enhanced. We have therefore provided point-by-point responses to the dignified comments raised as shown below and also highlighted all revised points in the manuscript in red.

Yours Sincerely,

Silas Adjei-Gyamfi

(Corresponding author)

**JOURNAL REQUIREMENT**

**Comment**: Please review your reference list to ensure that it is complete and correct. If you have cited papers that have been retracted, please include the rationale for doing so in the manuscript text, or remove these references and replace them with relevant current references. Any changes to the reference list should be mentioned in the rebuttal letter that accompanies your revised manuscript. If you need to cite a retracted article, indicate the article’s retracted status in the References list and also include a citation and full reference for the retraction notice.

**Response:** Thanks for this vital comment. The reference list is complete and correct. Our manuscript is free from any retracted papers or articles.

**REVIEWER #1**

**General comments:** I wish to congratulate the authors for a great job done in addressing the comments raised in the original manuscript. However, the authors are encouraged to address comment 8 of the previously mentioned- on the guiding principle for the number of study participants selected in the qualitative arm of the study. As rightly pointed out by the authors there is no specific formula for sample size determination in qualitative research except for the principle of data saturation. I think that the authors should state in the manuscript that data saturation was used as the guiding principle for recruitment. Once again congratulations!!!

**Response:** Many thanks for acknowledging the revisions made and commending the good efforts of the authors. We also appreciate your significant comments and deep comprehension of our manuscript which have greatly contributed to the improvement of our manuscript. As you suggested, a sentence has been added to take care of the principle of data saturation used in the qualitative arm of the study (see page 11; lines 221–222; methods and materials; sample size and sampling).

**REVIEWER #3**

**General comments:** Manuscript is interesting with an important but usually neglected cause of blood borne diseases. I recommend this manuscript theme and topic be published to increase the accessibility and expansion with regards to this topic in the future. The quantitative data is presented, but in a different available format, thus i am unable to review or comment with regards to the quantitative data used for the manuscript. The qualitative data is accessible via MP3. Manuscript is written in fairly good English, with only some correction and final proofreading required.

**Response:** Many thanks for reviewing our revised manuscript. We are grateful for your significant comments and deep comprehension of our manuscript which have greatly contributed to the improvement of our manuscript. The quantitative data is in Microsoft Excel. Furthermore, two different English-proficient persons have gone through the revised manuscript and have addressed the majority of the writing issues.

**Comment 1:** Author should define "beautician" and "barber" and explore why these 2 different profession were selected as the main subject of the study, and are they or are they not considered "high-risk workers"

**Response 1:** Thanks for this noble comment. Beauticians and barbers and their jobs have been described and/or defined in the manuscript (kindly see page 5; lines 97–103; introduction). In the present era especially in Ghana, the jobs of beauticians and barbers are interrelated. In the past years, beauticians only styled the hair of women but now they shave and style hair and also perform foot and nail care for all kinds of gender. Additionally, barbers who were known to only shave the hair of men are now shaving, styling, and sometimes providing nail care services to their customers irrespective of their gender. Geographically, people across the globe call them different names such as “beauticians”, “barbers”, “hairdressers”, “hairstylers” or “nail caretakers”. Ghanaians interchangeably label them as either “beauticians” or “barbers”. Meanwhile, the majority of them have no formal training in cosmetology and/or podiatry. This accounts for the reason why the authors sometimes label them as “local cosmetologists” in the manuscript.

Beauticians and barbers are at higher risk for blood-borne infections like hepatitis (especially B and C) because they provide services to a larger population without knowing the health status of these populations. These workers are religiously prone to occupational hazards such as being cut by used sharp tools like razor blades, and knives while or after attending to their customers. It therefore makes it very interesting to study about them and their job practices in the transmission of hepatitis infections. Compared to other street workers in Ghana like shoe repairers, food vendors/hawkers, and general goods hawkers among others, beauticians and barbers are at higher risk. Moreover, they are less recognized and minor groups where much attention has not been placed on them. Most of the focus and hepatitis-related studies are placed on maternal and child health (pregnant women, nursing mothers, neonates, etc) and commercial sex workers among other cohorts. Meanwhile, these workers are mostly ignored. On the other hand, it became necessary to find out whether these workers are trained on safety measures and whether their occupational practices are regulated or monitored.

**Comment 2:** Author should explain why a mixed method and triangulation analysis was used in this study, and how the qualitative data can add more impact to the findings.
**Response 2:** Thanks for the comment. As explained in the study design (kindly see page 9; lines 175–185), a mixed-method approach is vital in this study. According to Koopmans (2016), mixed methods is the preferable approach for addressing complex issues and exploring the linkage of factors operating at the individual, community, institutional, and societal levels. In the context of this study, little is known about the study, and using mixed methods was the best approach to describe the situation and provide answers to the study objectives. (Koopmans, M., 2016. Mixed Methods in Search of a Problem, Journal of Mixed Methods Research; Vol. 11, Issue 1, pp. 16 – 18. DOI: 10.1177/1558689816676662). The quantitative method is very necessary to provide proven statistical data whereas the qualitative study offers the opportunity to provide meaning behind the numbers. On the other hand, the study was carried out concurrently. Thus, a parallel study of both quantitative and qualitative strands within the same study. Triangulation analysis of the survey, observations, and key Informant Interviews was done to form a joint interpretation of the data. The qualitative arm gave more explanation on why the unsafe occupational practices are predominant among the study participants as well as the poor knowledge level exhibited by these street workers. For example, the qualitative arm revealed that there is a lack of licensing, monitoring, and training for street beauticians and barbers. Hence the poor practices and knowledge are attributed to the lack of regulatory systems and training on safety measures for these street beauticians and barbers as indicated by the qualitative arm.

**Comment 3:** Author should define the category of findings such as cut-offs for high vs low awareness, good vs poor knowledge
**Response 3:** Thanks for the vital comment. Good or poor knowledge was determined by using the median cut-off point of 14 knowledge-related items as described in Table 2. This means that a knowledge-related score of < 7 was regarded as “poor knowledge” while a score of ≥ 7 was regarded as “good knowledge” (see Table 2; page 15).

As seen in Table 5 (page 25), majority of the study participants (88.2%) were aware or had ever heard of hepatitis virus infections. This indicates that most of the participants had a high awareness level about hepatitis infections. Awareness in this study is used as “realizing or knowing that something (e.g. Hepatitis infection) exists” according to Oxford Learner’s Dictionary (kindly see; <https://www.oxfordlearnersdictionaries.com/definition/english/awareness>). It can also mean “consciousness”, “mindfulness”, “attention”, “alertness” as explained by Merriam-Webster dictionary (<https://www.merriam-webster.com/dictionary/awareness>). Hence, the awareness in this study was not categorized into low or high but rather based on frequency proportions.

**Comment 4:** Author should also include the how the initial beautician/barber was chosen from the premise, especially if there was more than one
**Response 4:** Thanks for the significant suggestion. The first street barber or beautician who met the inclusion criteria was recruited by approaching him/her while providing services to a customer in the community (see page 10; lines 208–209; methods and materials; sample size and sampling).

**Comment 5:** Suggest make the keywords one word, and singular and include Ghana to make it more easily and wider reach and accessibility

**Response 5:** Thank you for the comment. Keywords have been made singular as much as possible and Ghana is already included as a keyword (kindly see page 4; lines 70–76: Keywords).
